# Gaming Motivation and Negative Psychosocial Outcomes in Male Adolescents: An Individual-Centered 1-Year Longitudinal Study

**DOI:** 10.3389/fpsyg.2021.743273

**Published:** 2021-12-01

**Authors:** Ling Wang, Jialan Li, Yuzhou Chen, Xuemei Chai, Yuman Zhang, Zihan Wang, Hong Tan, Xumei Gao

**Affiliations:** ^1^Faculty of Psychology, Southwest University, Chongqing, China; ^2^Key Laboratory of Cognition and Personality, Ministry of Education, Southwest University, Chongqing, China; ^3^Chongqing Youth Vocational & Technical College, Chongqing, China; ^4^Chongqing Lixin Vocational Education Center, Chongqing, China

**Keywords:** gaming, motives, subtype, mechanisms, longitudinal, individual centered

## Abstract

“Gaming motivation” is a useful concept to draw upon when considering inconsistencies in the effects of online gaming on psychosocial wellbeing. However, most prior studies that utilize it are cross-sectional and do not allow that individuals can be driven by multiple motives. The present study uses an individual-centered method to classify gaming motivation styles of male adolescents and longitudinally observes the relationship between gaming motivations and psychosocial outcomes. A total of 929 healthy, male, adolescent gamers were recruited in October 2019 and classified into “recreational” “achiever,” and “escaper” categories according to their baseline gaming motivations and self-esteem levels. Then, 1-year incidence rates of players and relative risks (RRs) of social withdrawal problems, anxiety/depression syndrome, and self-destructive/identity problems were assessed. Recreational players were found to have the lowest incidence of all the three psychosocial problems among the three categories, achievers only had a moderate risk of social withdrawal, compared to recreational players, while escapers showed a strong risk for social withdrawal, anxiety/depression, and self-destructive/identity problems, relative to recreational gamers. Overall, the different motivation subgroups were associated with different psychosocial problems. Both achievers and escapers were found to be maladaptive, but their psychosocial outcomes were different, a finding that provides further insight into the psychological mechanisms underlying these subgroups.

## Introduction

Online games have become a prevalent source of entertainment in recent years. In 2020, there were an estimated 2.7 billion gamers across the globe, with children and young adults (age<20 years old) accounting for 22% of this population (Statista, 2020). The impacts of online gaming on the psychosocial wellbeing of players are inconsistently reported. Studies focusing on the benefits of online gaming have found that it helps to regulate emotions (Reinecke et al., 2011), promote socialization with peers (Ewoldsen et al., 2012), and reduce external problems by strengthening executive control (Ewoldsen et al., 2012). That is, online gaming has been shown to be good for the psychosocial wellbeing of players. On the contrary, other studies have inferred that online gaming may displace socialization in the real world, leading to gaming disorder or psychiatric symptoms, such as anxiety and depression (Király et al., 2015; Rumpf et al., 2018) and could foster aggression or a tendency toward violence. Such results suggest that gaming elicits internal or external problems and negatively affects the psychosocial wellbeing of players. Many factors have been considered to try to understand these inconsistent findings, such as game genre, gaming experience, and gaming motivation styles (King et al., 2019). Established risk factors for problematic gaming include age and sex; according to a recent meta-analysis, young males (prevalence 6.8%) are five times more likely to develop problematic gaming than females (prevalence 1.3%) (Fam, 2018).

### Gaming Motivation Styles and Psychosocial Problems

“Gaming motivations” are behavior-specific factors that drive people to start and continue with gaming (Billieux et al., 2013; Brand et al., 2019). Different motives may lead to various cognitive, behavioral, and affective outcomes (Vallerand, 1997). Yee’s gaming motivation model (Yee, 2006a) laid a solid foundation for subsequent research on the large variety of gaming motivation styles. Yee (2006a) extracted 10 gaming motives based on 3,000 massively multiplayer online role-playing game (MMORPG) profiles of players, then further refined these into three broad types: (1) achievement motive (including advancement, mechanics, and competition), (2) social motive (such as socializing, relationships, and teamwork), and (3) immersion motive (such as discovery, role-playing, customization, and escapism).

Moreover, previous studies focusing on the relationship between gaming motivation styles and psychosocial outcomes have indeed found different gaming motives to be associated with different outcomes. Some gaming motives have been consistently associated with gaming disorders, such as advancement (T’ng and Pau, 2021) and escape motive (Bowditch et al., 2018), while others, such as the social motive, are seen to be beneficial to the physical and mental health of players, at least to a certain extent (Billieux et al., 2013). Elucidating the inconsistencies found between online gaming and psychosocial wellbeing through the lens of motivation is therefore crucial and necessary (Yee, 2006a).

“Advancement” is a subcomponent of achievement motivation. It refers to the desire for acquiring power, making rapid progress, and accumulating items of wealth or status in a game (Yee, 2006b). A recent study found that 89% (*n* = 104) of MMORPG players had high scores on the advancement subscale, and they also had increased gaming time (Suznjevic and Matijasevic, 2010). The correlation between advancement motivation and gaming-related problems varies from 0.31 to 0.55 (Cross, 2016; Schimmenti et al., 2017). Players primarily motivated by achievement have been found to be lacking in premeditation and perseverance, to be sensation-seeking, and to possess only moderate self-esteem (Billieux et al., 2015). Their negative affective symptoms are second only to those of escapers (i.e., gamers predominantly motivated by escapism) (Billieux et al., 2015). Advancement motivation may also be associated with a cognitive distortion of in-game achievement (Molesworth and Mardon, 2015). However, advancement motivation might not always be problematic. For example, Jeng and Teng (2008) found that achievement-motivated players exhibited some positive personality traits, such as agreeableness.

The escapism motive, a subcomponent of immersion motivation, corresponds to the extent to which a gamer temporarily avoids, forgets, and escapes from real-life stresses or problems while in the virtual world (Yee, 2006b). Dauriat et al. (2011) reported the lowest association between the escapism motive and problematic gaming, whereas Laconi et al. (2017) reported the highest association (*r* = 0.555). A recent meta-analysis has calculated the overall association between advancement motivation and gaming-related problems to be 0.433 (O’Brien, 2019). Overall, multiple regression analyses have shown that the escapism motive could explain 31% (Yee, 2006a) to 46% (Kuss et al., 2012) of the total variance regarding problematic gaming. Similar to the advancement motive, players with a prominent escapism motive lack premeditation and perseverance but tend to act rashly in both negative and positive emotional contexts (Billieux et al., 2015). Escapers have very low self-esteem and do not like sensation-seeking (Billieux et al., 2015). They also have severer negative affective symptoms and slighter positive affective symptoms than achievers (Billieux et al., 2015). In addition, escapism is often comorbid with depression and high stress (Ballabio et al., 2017). A recent study found that players with stronger escapism motives have a more powerful sense of the realism of a virtual game, which might decrease their offline support with respect to real-world problems and harm their wellbeing (Kaczmarek and Drążkowski, 2014).

A number of limitations exist among current studies exploring the relationship between gaming motivation and psychosocial outcomes. First, most of these studies are of a cross-sectional design, which cannot be used to infer causality. Second, many use a variable-centered method, which is insufficient to understand the whole picture of gaming behavior because, according to the uses and gratification theory (Griffin, 2002), an individual may be simultaneously driven by multiple motives. Latent profile analysis (LPA), an individual-centered mixture modeling analysis based on model fit evaluation, can be applied to understand whole profiles of individuals by modeling latent classes (Oberski, 2016). The basic assumption of LPA is that heterogeneous variables can be explained by homogeneous and mutually exclusive categorical latent variables or clusters (Collins and Lanza, 2009). The LPA process includes three general steps: input variable determination, data preparation, and modeling.

### The Current Study

The present study was designed to investigate the longitudinal relationship between gaming motivation styles and negative psychosocial outcomes of male adolescents from an individual-centered perspective. Based on Yee’s gaming motivation model (Yee, 2006a) and existing findings, we elected to use *advancement*, *relationships*, and *escapism* as representative emotional factors of our research. In addition, we chose *social withdrawal*, *anxiety/depression syndrome*, and *self-injury/identification problems* as the indicators of psychosocial outcomes, for several reasons. First, these problems are typical and common internalization or externalization problems among young people. Second, they have been found to positively relate to both gambling disorders and substance use disorders (Chan et al., 2008; Terrone et al., 2018). We considered *self-esteem* as a moderator variable. Self-esteem is a subjective evaluation of people of their own worth (Niederhoffer and Pennebaker, 2002), as it can moderate the motivation and wellbeing of players (Goh et al., 2019) and strengthen the sense of value for those with a larger gap between the real-self and ideal-self during virtual-world immersion (Przybylski et al., 2012).

The purpose of the present research was two-fold: (1) to explore the subgroups of gaming motivation of male adolescents through LPA and (2) to longitudinally assess the risk of negative psychological outcomes in different motivation subgroups. Based on the subgroups previously identified by Billieux et al. (2015), this study set out to test four hypotheses, as follows.

**Hypothesis 1.** A total of four clusters will be identified.

Within this hypothesis, regarding achievers, a “cluster” will be characterized by a prominent achievement motive with moderate escapism motive, relationships motive, and self-esteem levels. For escapers, a cluster will be characterized by a prominent escapism motive with moderate achievement motive, relationships motive, and a low self-esteem level. A further cluster—hardcore players—will be characterized by prominent achievement and escapism motives simultaneously, but self-esteem and the relationship motive will be moderate. Finally, the cluster for recreational players will be characterized by low motivation levels with a moderate level of self-esteem.

Hypothesis 1 thus partly follows the study by Billieux et al. (2015), in which players were clustered into three “problematic” subgroups (i.e., achievers, escapers, and hardcore players) and two “non-problematic” subgroups (i.e., recreational players and social role-players). Problematic subgroups exhibit a higher-than-average negative outcome severity, whereas the non-problematic subgroups exhibit a lower negative outcome severity than average. Billieux et al. (2015) also found that problematic subgroups might be based on high impulsivity and low self-esteem.

Extending Hypothesis 1, Hypotheses 2–4 of the present study attempt to test the longitudinal relationship between gaming motivation and negative psychosocial outcomes in male adolescents, with a general assumption that problematic subgroups will develop more severe negative psychosocial outcomes than non-problematic subgroups.

**Hypothesis 2.** Achievers, escapers, and hardcore players will have a higher incidence of social withdrawal compared with recreational players.
**Hypothesis 3.** Achievers, escapers, and hardcore players will have a higher incidence of anxiety/depression syndrome compared with recreational players.
**Hypothesis 4.** Achievers, escapers, and hardcore players will have a higher incidence of self-injury/identification problems compared with recreational players.

## Materials and Methods

### Sample and Process

**FIGURE 1:**
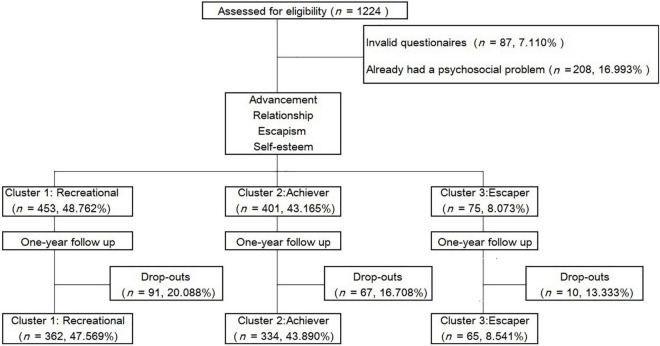
Study flow chart.

This was a 1-year longitudinal study. As young males are the vulnerable population within problematic gaming (Fam, 2018), our study invited 1,224 male MMORPG players in the first year of two vocational schools in southwest China and screened them using an electronic questionnaire in October 2019. We excluded 87 (7.11%) invalid questionnaires (completed by participants who finished the survey in a very short time or provided contradictory answers) and 208 (16.993%) participants who already met the criteria of the psychosocial problems, as measured by the 1991 Youth Self Report (YSR; 173 with social withdrawal problems, 154 with anxiety/depression, and 129 with self-injury/identification problems). We did not consider excluding internet gaming disorder because there is a considerably high overlap between maladaptive gaming motivation and internet gaming disorder. Ultimately, 929 participants (mean age = 15.950 ± 0.685 years) were included. At the baseline (T1), they were grouped into three clusters according to their gaming motivation and self-esteem (the modeling process is described in the Data Analysis section below). After 1 year (T2), a total of 761 (mean age = 16.954 ± 0.633 years) participants completed the follow-up questionnaire. Longitudinal research is inevitably affected by subject drop-out and/or missing values, and we decided to delete the entire case if missing values were found. A total of 168 (18.084%) participants were removed from the study: 23 (3.022%) through invalid questionnaires and 145 (19.053%) with questionnaires featuring partial or missing data. The numbers of drop-outs from Cluster 1 to Cluster 3 were 91 (20.088%), 67 (16.708%), and 10 (13.333%), respectively. These drop-out rates were considered acceptable. One of the main reasons for drop-out was that many students changed or forgot their ID used at the baseline survey, which resulted in a failure to match their questionnaires. A flow chart of the process is shown in Figure 1.

This study followed the tenets of the Declaration of Helsinki and was performed after obtaining ethical approval from the Faculty of Psychology, Southwest University. We acquired consent from the parents and teachers of the participants and the subjects themselves before starting the survey. To collect reliable data, we used their social messaging software (QQ) ID as the only identification code and guaranteed confidentiality and anonymity. Participants received incentives, such as small gifts (e.g., nail clippers).

### Measures

#### Gaming Motivation Styles

We used the Chinese Gaming Motivation Scale-39 (GMS-39) (Zhang et al., 2013) to measure the gaming motivation styles of players. This scale was originally developed by Yee (2006a) and includes 39 items—for example, “How important is it for you to level up as fast as possible?”—with each item rated according to a 5-point Likert scale (e.g., 1 = never, 5 = always) to measure the motivational level. Three overarching motives and 10 submotives were measured: (1) *achievement* (encompassing advancement, mechanics, and competition); (2) *social* (incorporating socializing, relationships, and teamwork); and (3) *immersion* (such as discovery, role-playing, customization, and escapism). A higher score indicated stronger motivation. The Chinese version of the GMS-39 has been found to have good validity and reliability (Zhang et al., 2013). In our study, subscales of advancement (a component of the achievement motive that has shown strong predictiveness of gaming-related negative outcomes (O’Brien, 2019), relationships, and escapism were retained. Cronbach’s alphas at T1 were 0.851, 0.713, and 0.632, respectively.

#### Psychosocial Outcomes

We adopted the social withdrawal problems, anxiety/depression syndrome, and self-destructive/identity problem subscales of the 1991 YSR (Huang et al., 2005) as the psychosocial outcome measurements of the present study. The YSR was developed by Achenbach (1991) and is one of the most commonly used instruments for measuring pediatric mental health worldwide. It has 112 items, such as “I would rather be alone than with others.” Each item is measured according to a three-level scale (0 = never, 1 = sometimes, and 2 = often). The scale has good reliability and validity (Huang et al., 2005). *T* scores were calculated according to the manual, and then diagnoses were made according to the cutoffs suggested by the authors of the scale (i.e., individuals with the top 2% of *T* scores were considered “positive” for the corresponding problems) (Huang et al., 2005). In the present study, Cronbach’s alphas for the social withdrawal, anxiety/depression, and self-destructive/identity problem subscales at T1/T2 were 0.733/0.776, 0.889/0.899, and 0.804/0.808, respectively.

#### Self-Esteem

We used the Rosenberg Self-Esteem Scale to measure self-esteem (Rosenberg, 1965). It has 10 questions (e.g., “At times, I think I am no good at all”), and each question is answered with reference to a 4-point Likert scale ranging from *strongly agree* to *strongly disagree* (five questions are reverse-scored). The higher the score, the higher the self-esteem level is measured to be. The Chinese version has shown high reliability and validity (Ji and Yu, 1993). Question 8 from the original scale was removed (based on the suggestion of the author), due to cultural differences. In the present study, Cronbach’s alpha at T1 was 0.832.

### Data Analysis

We used LPA to classify the players. First, input variables were selected, based on two principles: (1) parsimony (clusters comprising too many variables are difficult to explain) and (2) existing literature. In this study, motives for achievement, relationships, and escapism in the GMS-39 were selected to represent the main driving factors of youth game playing—these motives were also the most prominent at baseline. Self-esteem was included as a covariable.

Second, we undertook the data preparation. To make sure that each variable would contribute equally to the formation of the clusters, *Z* scores of all input variables were calculated to share the same metric.

The next, third step comprised modeling and evaluation. We used the Akaike information criterion (AIC), Bayesian information criterion (BIC), and adjusted BIC (aBIC) (Schwarz, 1978; Akaike, 1987; Sclove, 1987) to evaluate the model (see Table 1). The smaller the values of these three indicators, the better the model fit was deemed to be. When the sample size of the smallest cluster is>50, aBIC is the most accurate indicator (Yang, 2006). The *p*-Vuong–Lo–Mendell–Rubin likelihood ratio test (VLMR) (Vuong, 1989) was used to compare models. We calculated the incidence rates and 95% CIs of negative psychosocial outcomes for each cluster and performed chi-square tests to compare differences. *Post hoc* testing was conducted with *p*-values adjusted using the Holm–Bonferroni method. If *post hoc* test results were statistically significant, then relative risk (RR) was estimated using the robust error variance-modified Poisson regression method (specifically, the log link function) (Zou, 2004). RR is the ratio of the probability of an outcome in the exposed group to the probability of an outcome in the unexposed group. It is widely used as the effect size of the risk in epidemiological studies and can be explained intuitively (Agresti, 2011). The RR value also indicates whether the factor is a protective factor (RR < 1), irrelevant factor (1.0 < RR < 1.1), weak risk factor (1.2 < RR < 1.4), moderate risk factor (1.5 < RR < 2.9), strong risk factor (3.0 < RR < 9.9), or very strong risk factor (10 < RR) (Monson, 1990). The significance level was set at 0.05. Statistical softwares used were Mplus 8.0 (Muthén & Muthén, Los Angeles, CA, United States) and Stata^®^ 15.1 (StataCorp, College Station, TX, United States).

**TABLE 1:** Model evaluation.

Cluster number	AIC	BIC	aBIC	*p*-VLMR	Entropy
1	–	–	–	–	–
2	10,349.49	10,412.34	10,371.05	<0.001	0.863
3	10,191.30	10,278.31	10,221.15	0.0011	0.732
4	10,135.54	10,246.73	10,173.68	0.1706	0.713

*AIC, Akaike information criterion; BIC, Bayesian information criterion; aBIC, adjusted Bayesian information criterion; VLMR, Vuong–Lo–Mendell–Rubin likelihood ratio.*

## Results

### Clusters

As the number of clusters was increased, the aBIC continued to decrease; but, after adopting the three-cluster scheme, the decrease in aBIC was very small. The *p*-VLMR is an index with which to compare differences between models. If the *p*-VLMR is>0.05, it means that the *k* clusters scheme is not better than the *k* – 1 clusters scheme. In our study, four clusters were not better than three clusters (*p* = 0.1706). Entropy is an index of classification accuracy, but accuracy will reduce with a larger sample size. Information may be lost with more clusters, so we decided that the three clusters scheme was optimal for this study (as detailed in Table 1).

### Clustering Characteristics and Naming

**FIGURE 2:**
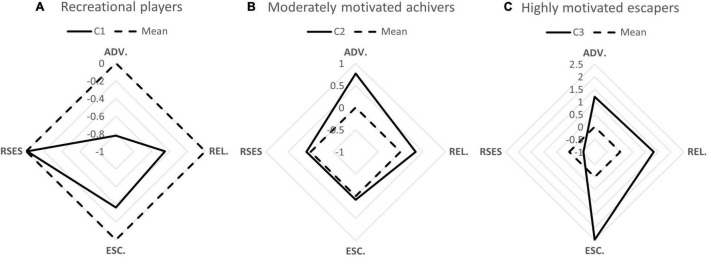
Radar map of characteristics for each cluster. ADV, advancement motive; C, cluster; ESC, escapism motive; REL, relationship motive; RSES, Rosenberg Self-Esteem Scale. Full lines indicate mean *Z* scores for each cluster, dashed lines denote the mean *Z* score of the entire sample.

The characteristics of each cluster are shown in Table 2 and Figure 2. Cluster 1 (C1 in Figure 2) had a total of 453 (48.762%) individuals. The gaming motivations of participants in C1 were lower than the overall average, but self-esteem levels remained moderate. Their weekly gaming time at T1 was 10.191 ± 8.491 h, and they were designated as *recreational players* (Figure 2A). Cluster 2 (C2) had a total of 401 (44.163%) individuals with moderate to strong achievement motives, slightly higher than average relationship motives, and moderate self-esteem. Their weekly gaming time at T1 was 14.306 ± 9.933 h, and C2 was termed the *achiever* subtype (Figure 2B). There were 75 (5.617%) individuals in Cluster 3 (C3), which was characterized by strong escapism motives, middle to strong achievement, middle to strong relationship motives, and lower than average self-esteem. The weekly game time at T1 was 19.647 ± 10.916 h, and the subtype was termed *escapers* (Figure 2C). Gaming time changes between T1 and T2 were not significant (paired *t-*tests, all *p* > 0.05), but they were different among the three groups (variance test, *post hoc* test, all *p* < 0.05).

**TABLE 2:** Baseline characteristics by subgroup.

	Overall mean	Recreational^a^	Achievers^b^	Escapers^c^
Advancement (1–5)^d^	2.8251.045	1.9470.527	3.5910.618	4.0330.736
Relationships (1–5)^d^	2.1810.822	1.7950.598	2.4240.743	3.2090.997
Escapism (1–5)^d^	2.0060.796	1.6890.542	2.0230.575	3.8220.638
Self-esteem (0–4)^d^	2.940.485	2.9390.467	2.990.494	2.6730.464
Gaming hours, T1^e^	12.739.759	10.1918.491	14.3069.933	19.64710.916
Gaming hours, T2^e^	12.6259.745	10.7518.953	13.8119.909	16.96210.88

*^a^n = 453.*

*^b^n = 401.*

*^c^n = 75.*

*^d^Numbers in parentheses are the range of the Likert scale.*

*^e^T represents time point.*

### Cross Time Validation: Player Subtype and Social Withdrawal, Anxiety/Depression Syndrome, and Self-Destructive/Identity Problems

The incidences of withdrawal problems, anxiety/depression syndrome, and self-destructive/identity problems among the clusters were significantly different at T2 (see Table 3). Regarding withdrawal problems, the incidence rates were 5.249% (95% CI [2.94, 7.557]), 11.377% (95% CI [7.95, 14.8]), and 16.923% (95% CI [7.56, 26.286]) in the recreational, achiever, and escaper subgroups, respectively. Achievers were 2.168 (95% CI [1.275, 3.685]) times more likely to develop social withdrawal than recreational players, while the escapers were at 3.224 (95% CI [1.609, 6.457]) times more at risk of social withdrawal than recreational players. There was no significant difference in social withdrawal between achievers and escapers.

**TABLE 3:** One-year incidence rates and RRs of negative psychosocial outcomes by subgroup.

		Recreational		Achievers		Escapers		Intergroup overall test		*Post hoc* test	
						
		*n*	% (95% CI)	*n*	% (95% CI)	*n*	% (95% CI)	χ^2^ (*df* = 2)	*p*	Results^#^	RR (95% CI)
Social withdrawal	T1	0	0.000%	0	0.000%	0	0.000%			b>a	2.168 [1.275, 3.685]
	T2	19	5.249% [2.940, 7.557]	38	11.377% [7.954, 14.8]	11	16.923% [7.56, 26.286]	13.591	0.001*	c>a	3.224 [1.609, 6.457]
Anxiety/depression	T1	0	0.000%	0	0.000%	0	0.000%			c>a	4.376 [2.078, 9.215]
	T2	14	3.867% [1.872, 5.863]	18	5.389% [2.955, 7.823]	11	16.923% [7.56, 26.286]	17.695	0.001*	c>b	3.140 [1.556, 6.338]
Self-destructive/identity	T1	0	0.000%	0	0.000%	0	0.000%			c>a	5.198 [2.635, 10.252]
	T2	15	4.144% [2.081, 6.206]	21	6.287% [3.671, 8.904]	14	21.538% [11.273, 31.804]	27.240	0.001*	c>b	3.425 [1.837, 6.384]

*T, time point; CI, confidence interval; RR, relative risk. Values in square brackets indicate the 95% CIs (upper and lower limits).*

*p < 0.05.

*^#^p-value was Holm–Bonferroni adjusted (Aickin and Gensler, 1996).*

Regarding the anxiety/depression facet, incidence rates for recreational players, achievers, and escapers were 3.867% (95% CI [1.872, 5.863]), 5.389% (95% CI [2.955, 7.823]), and 16.923% (95% CI [7.56, 26.286]), respectively. Escapers were 4.376 (95% CI [2.078, 9.215]) times as likely as recreational players to experience anxiety/depression syndrome. Escapers were also at greater risk of anxiety/depression syndrome compared with achievers.

As regards self-destructive/identity problems, the respective incidence rates were 4.144% (95% CI [2.081, 6.206]), 6.287% (95% CI [3.671, 8.904]), and 21.538% (95% CI [11.273, 31.804]). Escapers had a 5.198-fold risk (95% CI [2.635, 10.252]) of self-destructive/identity problems compared with recreational players, and they were also at greater risk than achievers.

## Discussion

We set out to explore the relationship between gaming motivation style and various negative psychosocial outcomes for gamers. A total of 929 male adolescents participated in this 1-year (October 2019–October 2020) study, which featured two time points. We identified three motivation subgroups (recreational, achievers, and escapers), and differences in the incidences of social withdrawal, anxiety/depression syndrome, and self-destructive/identity problems in each subgroup were assessed during the 1-year follow-up. The results preliminarily suggest that different gaming motivation subgroups are associated with different psychosocial outcomes.

### Cluster Characteristics

Regarding the number of clusters, we found three, partially supporting Hypothesis 1. We did not find hardcore player subgroups in the present sample, which is inconsistent with previous research findings (Billieux et al., 2015). One tentative explanation is that our sample comprised adolescent students who spend most of their time on classroom learning. Thus, they have shorter game exposure and less game experience than adults do. At the early stage of exposure, the motivation of individuals is often very simple; they are commonly driven by positive rewards (Brand et al., 2019) or fun-seeking (Tian, 2017). With greater exposure, their motivations become more complex—such as compulsion, at the late stage of addiction (Everitt and Robbins, 2005; Koob and Volkow, 2010; el-Guebaly et al., 2012). Accordingly, the hardcore player subgroup might not exist in this study’s population. As for the lack of a social player subgroup, the reasons for this might be that our sample was made up of male adolescents, and previous studies have found male players to have less relationship motivation than female players (Yee, 2006a).

With reference to the characteristics of subgroups, our results support Hypothesis 1. We found that recreational players mainly showed moderate levels of self-esteem, low motives for online relationships and for gaming, and they also had the shortest game time among all three subgroups, which is similar to the findings of a previous study (Billieux et al., 2015), and this subgroup was regarded as being adaptive. We found that escapers are mainly driven by escapism and have low self-esteem, which is consistent with previous findings (Billieux et al., 2015). Escapers spent most of the time on gaming among the three subgroups of our study. Achievers had moderate self-esteem and a prominent achievement motive. However, our results are different from those of Billieux et al. (2015), in that achievers exhibited an average level of escape motive. The reason for this variation may be that the sample in our study consisted of high school freshmen who were experiencing a changing environment. Arguably, they faced more real-world problems, which increased their escapism trend. Another difference between the two studies is that both achievers and escapers were found to have higher than average relationship motives, which might be because our sample comprised young boys. They are facing socialization tasks at this age and as a result, give more importance to peer relationships than people of other life stages do.

### Gaming Motivation Styles and Social Withdrawal

Compared with recreational players, the risk for achievers and escapers of developing social withdrawal was found to be moderate and strong, respectively, but differences between achievers and escapers were not significant statistically. Hypothesis 2 was thus partially supported.

Regarding the association between achievers and social withdrawal, a tentative explanation may be that achievers, having medium self-esteem and being mainly motivated by in-game achievements, might believe that success in the virtual world is a manifestation of their true ability, which weakens their interest in pursuing success in the real world and ultimately leads to social retreat. This inference is in line with two prior studies that showed that players feel more achievement in virtual worlds than in reality (Ko et al., 2005; Yee, 2006a), which might render achievement in the real world less attractive. In other words, to these gamers, it seems as if “virtual” achievement overwhelms any real-world achievement, suggesting a potential denial of real life and an over-involvement with virtual life.

The risk of social withdrawal was found to be much higher for escapers than for recreational players. Escapers, having low self-esteem and mainly motivated by an escapism motive, tend to use online games to avoid real problems or negative feelings. Online games can make them experience self-worth in the virtual world, forget real problems, and temporarily adjust negative emotions. In doing so, they “dissociate” from real life rather than adapt to society (Schimmenti and Caretti, 2010), but the real-life problems may not be resolved, instead might worsen over time, which will bring the individual more pressure and eventually cause social withdrawal. The stronger online relationship motive of these gamers could be seen as compensation for their social withdrawal from reality. In short, the stronger the motive to escape reality, the more risk there is for social withdrawal. Social withdrawal is a typical internalization problem of adolescents (Bo-Liang and Lei, 2004), but investigations into the relationship between gaming motivation and social withdrawal are rare.

### Gaming Motivation Styles and Anxiety/Depression Syndrome

Our research found that only escapers have a risk of developing anxiety/depression syndrome, compared to recreational players, and the effect size was strong. These results partially support Hypothesis 3.

An escapism motive is believed to be a response to negative affections or existing problems in real life (Schimmenti et al., 2012; Kardefelt-Winther, 2014), but our study excluded depression at baseline. Thus, we argue that escapism *per se* might lead to anxiety/depression problems. On the one hand, the self-esteem levels of escapers were the lowest in our sample. In a recent meta-analysis, low self-esteem has been proved to be a risk factor for depression and anxiety (Sowislo and Orth, 2013). On the other hand, escapism may be a manifestation of narcissism. One previous study found that the relationship between narcissism and gaming disorder was fully mediated via escapism (Wai Yen et al., 2020). Narcissism can increase the risk of depression in adolescents, based on guilty and shame (Anastasopoulos, 2007). We could infer that escapers may intend to utilize excessive gaming as a self-medication to cope with low esteem and feelings of guilt and shame. However, escapism and depression are theoretically reciprocal, and the dynamic relationship between the two constructs is worthy of further study. The differences between achievers and recreational players were not statistically significant, which may be because of the protective effect of self-esteem and the sense of accomplishment gained in a game. In summary, only escapers had an increased risk of anxiety/depression syndrome. As for achievers and anxiety/depression syndrome, we did not find a significant difference between achievers and recreational players. A possible reason for this is that achievers have moderate self-esteem and might experience more positive emotions than escapers, which sheltered them from anxiety/depression syndrome.

### Gaming Motivation Styles and Self-Destructive/Identity Problems

The risk of self-destructive/identity problems among escapers could be considered as being strong, compared to that for recreational players. Hypothesis 4 was therefore partially supported.

From the perspective of motivation, it is reasonable to infer that, because escapers have a stronger intention to try to escape when problems in reality are not serious, they use online gaming to relieve their emotions. When real problems are very serious and cannot be solved by oneself, though, escapism behavior may escalate into self-harm as a punishment for incompetence. This hypothesis is in line with the emotion regulatory model of self-injury that posits that people use self-injury behavior as a coping style to alleviate negative feelings or affective arousal, thereby regulating one’s emotions (Haines et al., 1995; Gratz, 2003). Problem gaming motivated by escapism and self-destructive/identity behaviors may have the same issue with different severities. To the best of our knowledge, no research has directly examined the relationship between gaming motivation styles and self-destructive/identity problems. Analyzing the self-destructive/identity problems of the achievers, we did not find a significant difference between achievers and recreational players. The reason for this may be similar to that given in the previous section, in that achievers might experience more positive emotions than escapers because they have higher self-esteem, and, as a result, there is no comparable need for emotional regulation.

### Clinical Significance

As can be seen from the results of this study, it is of diagnostic and intervention significance to analyze gaming motivation. Clinically, achievement-motivated or escapism-motivated players may exhibit similar symptoms, such as loss of control and craving. Analyzing the gaming motivation helps us to understand the etiology of gaming disorder and thereby increase the response rates to treatment. The negative outcome found to be caused by the achievement motive has primary features (Billieux et al., 2015), and patients in this subgroup might benefit from interventions focusing on forming an adaptive achievement cognition. Negative outcomes caused by the escapism motive are secondary to real-world or emotional problems. Patients in this subgroup might benefit from strategies enhancing their ability to cope with reality, solve problems, and regulate emotions.

### Limitations and Suggestions

This study had several limitations. First, we did not measure the gaming motivation at the 1-year follow-up. Gaming motivation may change with time, yet this study assumed that the motivation of an individual remains stable for a certain period and did not consider longitudinal changes.

Second, the baseline comparability of a longitudinal study had a very important impact on this study’s results. We excluded players who were already experiencing the specified problems, but natural vulnerability differs among individuals, and so heterogeneity at baseline cannot be ruled out. However, we did control for the two major factors affecting gaming: gender and age. Moreover, our large sample size (*N* = 929) helped balance the differences at baseline.

Third, the LPA method that we used may yield classification errors. However, as an individual-centric method, LPA overcomes the shortcomings (e.g., over-reductionism) of variable-centric analysis and has been widely used in different fields.

Fourth, we only included Chinese male adolescents aged 15–16; the impacts of different cultural backgrounds, sexes, and ages remain to be studied.

Fifth, the follow-up time interval was 1 year. However, it is possible that some endpoints appeared earlier, and this study would have failed to collect that information. Therefore, future research should shorten the follow-up time interval and use Cox proportional hazards regression models for the analysis.

Finally, the internal reliability for the escapism subscale was low. One probable explanation could be that the scale was not sufficiently suitable for students. For example, one question from this subscale is “How often do you play to relax from the day’s work?” Responses of students to this question might vary a lot, and decrease the internal reliability, because most of them do not need to work at this age. Instead, many of them might use gaming to escape pressure from the study; hence, the severity of escapism might be underestimated in this study.

## Conclusion

Gaming motivation styles are useful for understanding the heterogeneity in the effects of online gaming on the psychosocial wellbeing of players. This study identified three subgroups; recreational players suffered the least negative psychosocial outcomes, while achievers and escapers were found to have problematic tendencies, albeit with different negative psychosocial outcomes. Both achievers and escapers are at increased risk of social withdrawal, but only escapers also have increased risks of depression/anxiety syndrome and self-destructive/identity problems. Differences in negative psychosocial outcomes that indicate different mechanisms could also guide future psychological evaluations and public health interventions.

## Data Availability Statement

The raw data supporting the conclusions of this article will be made available by the authors, without undue reservation.

## Ethics Statement

The studies involving human participants were reviewed and approved by the Faculty of Psychology, Southwest University. Written informed consent to participate in this study was provided by the participants’ legal guardian/next of kin.

## Author Contributions

XG: conceptualization, funding acquisition, project administration, supervision, and writing—review and editing. LW: formal analysis, investigation, methodology, software, and writing—original draft. JL, YC, and XC: investigation, resources, data curation, and validation. YZ, ZW, and HT: resources, methodology, software, and visualization. All authors contributed to the manuscript and approved the submitted version.

## Conflict of Interest

The authors declare that the research was conducted in the absence of any commercial or financial relationships that could be construed as a potential conflict of interest.

## Publisher’s Note

All claims expressed in this article are solely those of the authors and do not necessarily represent those of their affiliated organizations, or those of the publisher, the editors and the reviewers. Any product that may be evaluated in this article, or claim that may be made by its manufacturer, is not guaranteed or endorsed by the publisher.
